# First Documented Pathologies in *Tenontosaurus tilletti* with Comments on Infection in Non-Avian Dinosaurs

**DOI:** 10.1038/s41598-019-45101-6

**Published:** 2019-06-18

**Authors:** T. C. Hunt, J. E. Peterson, J. A. Frederickson, J. E. Cohen, J. L. Berry

**Affiliations:** 1 Department of Biology, University of Oklahoma, Norman, Oklahoma 73072 USA; 2 Sam Noble Oklahoma Museum of Natural History, 2401 Chautauqua Avenue, Norman, Oklahoma 73072 USA; 3 Department of Geology, University of Wisconsin-Oshkosh, 800 Algoma Blvd, Oshkosh, Wisconsin 54901 USA; 4 Department of Biological Sciences, Southwestern Oklahoma State University, 100 E Campus Drive, Weatherford, Oklahoma 73096 USA; 5 Department of Biology, Loyola Marymount University, 1 Loyola Marymount University Drive, Los Angeles, California, 90045 USA; 6 University of Oklahoma Health Sciences Center, Department of Medical Imaging and Radiation Sciences, 1200 North Stonewall, AHB-3021, Oklahoma City, OK 73117 USA

**Keywords:** Palaeontology, Infection

## Abstract

In 2001, a nearly complete sub-adult *Tenontosaurus tilletti* was collected from the Antlers Formation (Aptian-Albian) of southeastern Oklahoma. Beyond its exceptional preservation, computed tomography (CT) and physical examination revealed this specimen has five pathological elements with four of the pathologies a result of trauma. Left pedal phalanx I-1 and left dorsal rib 10 are both fractured with extensive callus formation in the later stages of healing. Left dorsal rib 7 (L7) and right dorsal rib 10 (R10) exhibit impacted fractures compressed 26 mm and 24 mm, respectively. The fracture morphologies in L7 and R10 indicate this animal suffered a strong compressive force coincident with the long axis of the ribs. All three rib pathologies and the pathological left phalanx I-1 are consistent with injuries sustained in a fall. However, it is clear from the healing exhibited by these fractures that this individual survived the fall. In addition to traumatic fractures, left dorsal rib 10 and possibly left phalanx I-1 have a morphology consistent with post-traumatic infection in the form of osteomyelitis. The CT scans of left metacarpal IV revealed the presence of an abscess within the medullary cavity consistent with a subacute form of hematogenous osteomyelitis termed a Brodie abscess. This is only the second reported Brodie abscess in non-avian dinosaurs and the first documented occurrence in herbivorous dinosaurs. The presence of a Brodie abscess, known only in mammalian pathological literature, suggest mammalian descriptors for bone infection may be applicable to non-avian dinosaurs.

## Introduction

In recent years, the field of dinosaurian paleopathology has grown significantly, expanding the breadth of taxa involved and types of pathologies reported^1–8^. Through this growing data set it has become clear that while injuries or abnormalities are well-documented, demonstrable infection is not common in dinosaurs^9^. In a review of 119 theropod pathologies, Molnar^10^ found only seven instances of infection. However, some large theropod skeletons (e.g., *Allosaurus fragilis*^4,11^ and *Tyrannosaurus rex*^10^) show multiple isolated cases of infection. In contrast, herbivorous dinosaurs typically only display single cases of infection^7,12,13^ with rare instances of multiple infected elements^8,14^. Additionally, it has been suggested that non-avian dinosaurs did not exhibit hematogenous (blood-borne) osteomyelitis, due to the inferred similarities in their immune response with reptiles and their apparent ability to isolate osteomyelitis to single elements^4^.

Here, we report five skeletal pathologies on a nearly complete subadult *Tenontosaurus tilletti*, OMNH 58340 (Sam Noble Oklahoma Museum of Natural History, Norman, OK, USA), from the Antlers Formation of southeastern Oklahoma. Prior reports of bone modification on *T*. *tilletti* skeletons include only feeding traces attributed to the predatory theropod *Deinonychus antirrhopus*, none of which contain evidence of healing^15,16^. The pathological bones observed in this study include: left dorsal ribs 7 and 10, right dorsal rib 10, left pedal phalanx I-1, and left metacarpal IV. The pathologies present in this individual represent two, possibly three distinct cases of infection, including the first documentation of a Brodie abscess in an ornithischian dinosaur.

## Materials and Methods

OMNH 58340 is a subadult *Tenontosaurus tilletti* collected from OMNH locality V821, during the 2000–2001 field season^17^. V821 is located in Atoka County, Oklahoma, within the Aptian-Albian aged Antlers Formation^18^. In addition to OMNH 58340, the articulated vertebrae of the holotype specimen for *Sauroposeidon proteles*^19^ was collected 3–5 m stratigraphically below OMNH 58340 at V821^20^. OMNH 58340 is nearly complete and articulated with only the mid-section of the tail missing due to weathering prior to discovery.

In total, OMNH 58340 has five pathological elements: two left ribs (L7 and L10), a right rib (R10), left pedal phalanx I-1, and the left metacarpal IV. Bone abnormalities were diagnosed through macroscopic inspection and with the aid of computed tomography (CT). Pathologies were classified as traumatic (from injury), infectious (from viral, bacterial, and fungal foreign agents), traumatic-infectious (infection following an injury), or developmental (caused by growth disturbance during development) sensu Hanna^11^.

CT scans were performed at the University of Oklahoma Health Science Center using a Philips Brilliance 16 slice CT scanner. Metacarpal IV was scanned at 120 kVp and 151 mAs at 16 × 0.7 mm slices and reconstructed at 0.35 mm overlapping slices with a high-resolution filter on a 512 matrix. Phalanx I was scanned at 120 kVp and 251 mAs at 16 × 0.7 mm slices and reconstructed at 0.35 mm overlapping slices with a high-resolution filter on a 512 matrix. Ribs L7–L10, and R10 were scanned at 140 kVp and 199 mAs at 16 × 1 mm slices and reconstructed at 0.5 mm overlapping slices with a high-resolution filter on a 512 matrix. Data were exported as DICOM files representing a 69.0 mm field of view. The CT scans generated during this study are available as TIFF files at figshare.com, 10.6084/m9.figshare.6463169. Measurements of the circumference of phalanx-1 were taken macroscopically with a measuring tape, and endosseous structures were measured on the CT scans using the measure function in Fiji^21^.

## Results

### Phalanx I-1

**Figure 1:**
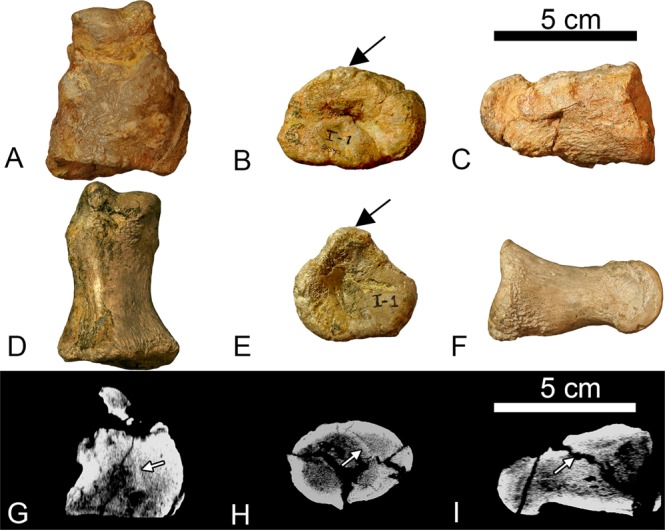
OMNH 58340 left pedal phalanx I-1 (**A**–**C**, **G**–**I**) and non-pathological right pedal phalanx I-1(**D**–**F**) for comparison. (**A**,**D**,**G**) dorsal view. (**B**,**E**,**H**) proximal view. (**C**,**F**,**I**) lateral view. The rugose callus has thickened the diaphysis and drastically changed the shape of the proximal articular surface. The black arrows denote the location of the extensor tubercle and the degree to which callus growth on the pathological specimen has obscured or remodeled the tubercle. The white arrows in (**G**,**H**) indicate original bone cortex surrounded by a mottled layer (woven bone) and a thin solid white layer (lamellar bone) this morphology is consistent with a callus in the process of remodeling. The white arrow in **I** shows the location of a large taphonomic fracture. The rough outer texture of the callus indicates that this element was secondarily affected by a non-suppurative infection (traumatic-infectious).

The left pedal phalanx I-1 has a large, irregularly shaped expansion surrounding the diaphysis and the proximal articular surface (Fig. 1A–C). The periosteal surface is rugose and covered in shallow, irregular pits with multiple areas of raised bony ridges, primarily located on the inferior aspect of the lateral side (Fig. 1C). The expansion has increased the circumference of the diaphysis to nearly equal the circumference of the proximal articular surface. The inflation of the diaphysis is not uniform; the dorsal side shows more expansion than the plantar side, particularly on the dorsomedial edge (Fig. 1H). The expansion surrounding the diaphysis at its widest extent has a circumference of 14.4 cm, whereas the unaffected right phalanx has a circumference of 8.0 cm. Proximal to the distal condyle on the dorsal side, there is a pronounced lip terminating the extent of the expansion, leaving the distal condyle unaffected. Deformation caused by the expansion modified the proximal articular surface into a wide, ovate shape with no observable extensor tubercle (Fig. 1B). In contrast, the proximal articular surface of the contralateral phalanx is pentagonal in outline (Fig. 1E). Additionally, the shape of the fossa within the proximal articular surface of phalanx I-1 has been deformed from circular in outline to elliptical, and its depth has been elaborated by the growth of the expansion (Fig. 1B,E).

CT scans reveal the differences in relative density between osseous tissue. Lucent (dark) areas represent low-density bone such as cancellous or reactive woven bone, whereas sclerotic (white) areas represent higher density bone, such as cortical bone^22^. The CT scans of OMNH 58340 reveal a faint, internal, dense line observed one-third of the way down the shaft from the proximal end, suggesting the presence of the original diaphyseal cortical bone (Fig. 1G,H). When outlined, the internal line conforms to the corresponding section of the diaphysis in the unaffected phalanx. The cortical bone can also be observed in the proximal view and is present in the proximal two-thirds of the element. Based on the location of the original cortical bone, the primary direction of pathological growth was in the dorsomedial direction. Additionally, the CT scans reveal this element to be taphonomically fractured to a degree atypical of other bones in this specimen. The fracturing present on the phalanx crosscuts pathological features and therefore is most likely due to taphonomic processes (Fig. 1I). This could be the result of the pathology reducing the structural integrity of the phalanx. Bones adjacent to phalanx I-1 are unaffected, except for the distal condyle of metatarsal I. The proximal articular surface of left pedal phalanx I-1 is more concave than the unaffected contralateral phalanx I-1; therefore, the distal condyle of left metatarsal I was remodeled accordingly, resulting in a slightly more convex articular surface than that of the right metatarsal I.

#### Diagnosis

The pathological left phalanx I-1 of OMNH 58340 bears a strong resemblance to that of two pathological pedal phalanges belonging to *Allosaurus fragilis*, described by Hanna^11^. The right pedal phalanx III-1 of MOR 693 (Museum of the Rockies, Montana State University, Montana, USA) and the left pedal phalanx III-1 of UUVP 1657 (University of Utah Vertebrate Paleontology, Salt Lake City, Utah, USA) from the Cleveland-Lloyd Quarry both possess a large osseous expansion covering the proximal two-thirds of the diaphysis and the proximal articular surface, similar to the abnormality on the left phalanx I-1 of OMNH 58340^11^. In the phalanges described by Hanna^11^, the diaphyseal and proximal articular expansions are interpreted as involucrum—a periosteal outgrowth of bone that surrounds the original bone in response to an infection within the medullary cavity—that may have occurred secondarily to a trauma-related fracturing of these elements. Therefore, a combination of callus formation and infection-related bone growth could have produced these abnormalities. Both MOR 693 and UUVP 1657 possess what Hanna^11^ interpreted to be penetrating lesions, cloacae for the drainage of pus produced in response to osteomyelitis, on the exterior of these phalanges. The penetrating lesions and involucrum are interpreted as evidence of infection and therefore Hanna^11^ diagnosed these pathologies as post-traumatic chronic suppurative osteomyelitis. Rega^9^ challenged this diagnosis suggesting that the phalanx I-1 of MOR 693 lacks cloacae and that the surface texture and inflation of the element could be indicative of a benign bone tumor called an osteochondroma. The pathologically affected regions of the left phalanx I-1 of OMNH 58340 similarly lacks cloacae. Considering what has been suggested for similar abnormalities on the aforementioned *Allosaurus* phalanges, a differential diagnosis of OMNH 58340’s phalanx I-1 should include consideration of three possible etiologies: an osteochondroma, callus formation, and osteomyelitis.

Osteochondromas are the most common bone tumors found in humans^23^. They are characterized as benign outgrowths that consist of cancellous and cortical bone capped by hyaline cartilage. The pathognomonic characteristic of this type of lesion is its continuity with the underlying non-pathological bone cortex^23^. Therefore, in diagnosing an osteochondroma it is crucial to determine if the expansion is in continuity with the cortical bone and underlying medullary cavity. The presence of the original cortical bone (Fig. 1G,H) within the expansion strongly argues against a diagnosis of an osteochondroma for this element because the expansion is not continuous with the original cortical and medullary cavity. Osteochondromas do not typically envelope the bone they outgrow from, rather this morphology is more consistent with either callus formation or osteomyelitis.

The latter etiologies are not mutually exclusive; callus formation can begin, and bacteria can be introduced secondarily causing osteomyelitis. The CT scans of left phalanx I-1 reveal multiple characters that suggest the proximal expansion of phalanx I-1 is related to callus formation following a fracture. The white arrows in Fig. 1G,H indicate the location of the original cortical bone of the diaphysis. Peripheral to the original cortical bone on the dorsomedial edge, there is a layer of woven bone, followed by denser bone characteristic of advanced remodeling^22^. Internally, there is no evidence of a malunion of the cortical bone within the phalanx; however, the pattern of bone tissues in the CT scans are typical of callus formation resulting from a fracture^22^. The lack of resolution surrounding original cortical bone may reflect a callus in the later stages of healing, where osteoclastic activity has begun resorbing the original cortical bone^22^. This inference may be corroborated by the fact that the left metatarsal I has a more convex distal articular surface, to match the increased concavity of the pathological proximal articular surface on the phalanx, a degree of remodeling that would indicate a significant span of time has passed. While lacking the cloaca described in the pathological *Allosaurus* phalanges, the left pedal phalanx of OMNH 58340 does exhibit a rough, pitted texture covering the callus that appears similar to the texture associated with osteomyelitis in a titanosaur sauropod tail from Anacleto Formation of Argentina, in which the periosteal surfaces of multiple caudal vertebrae were covered with a rough ‘microbubbly’ surface texture (Fig. 1C)^14^. Therefore, the rugose texture of the periosteal surface surrounding the phalanx may suggest secondary osteomyelitis, thus this pathology is considered traumatic, and possibly infectious^11^.

### Ribs

**Figure 2:**
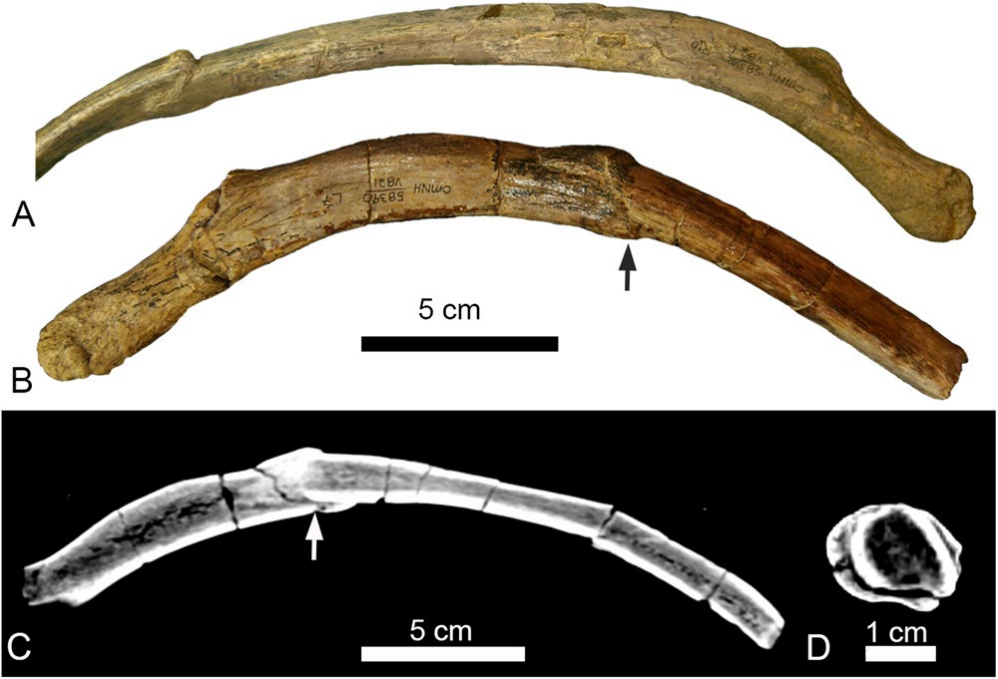
Fractured right dorsal rib (R10) (**A**) and fractured left dorsal rib (L7) (**B**–**D)**. (**A**) R10 in anterior view. (**B**) L7 in anterior view showing an impacted fracture (black arrow). (**C**) Anterior slice through L7 showing 24 mm of compaction and angulation of the distal rib element. (**D**) Cross-section of rib L7 in proximal view, location of slice indicated by the white arrow in (**C**). The orthogonal slice shows the stacked cortical bone resulting from the impaction of the distal rib element into the proximal element. The pathology is classified as traumatic.

Left dorsal rib 7 (L7) is fractured just below its greatest curvature, 75 mm distal to the tuberculum (Fig. 2B). The distal portion of the rib is compressed and telescoped approximately 14.5 mm proximally into the broken end of the proximal rib segment, resulting in a total shortening of 26 mm. The ventral side of the rib is cracked and bulged outward to accommodate the impaction of the distal rib segment. Surrounding the fracture and between the elements there is minimal callus formation, however the bones are fully fused. The CT scans (Fig. 2C,D) of this pathology clearly show stacked cortical bone on the ventral side of the rib.

Right dorsal rib 10 (R10) is fractured 140 mm below the tuberculum where the rib begins to straighten out. The distal end of the fracture is displaced 18 mm proximally (Fig. 2A), for a total shortening of the rib of 24 mm. While still an impacted fracture the morphology differs slightly from that of L7. There is minor angulation of the distal rib segment so that the anterior cortical bone of the distal rib segment overlays the anterior cortical bone of the proximal rib segment. Furthermore, some deformation and cracking is present on the ventral side like what is seen in L7. Like L7, there is minimal callus formation and the segments are fully fused.

**Figure 3:**
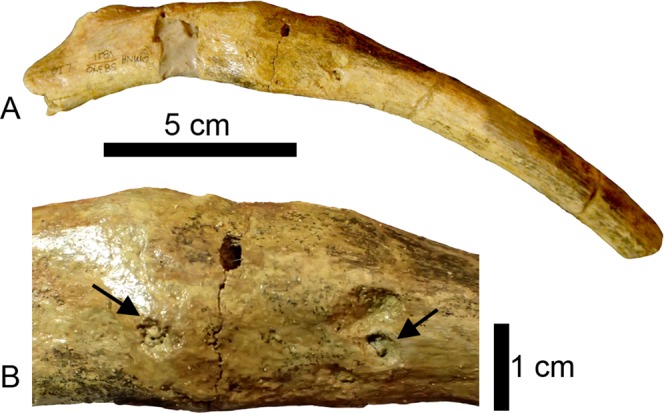
(**A**) Left dorsal rib 10 (L10) in anterior view showing a callus that has expanded the shaft of the rib. (**B**) Enlarged anterior view of callus surface showing eye-shaped callus margin with two lytic lesions (black arrows) present on the callus margin. These lesions are suggestive of osteomyelitis (traumatic-infectious).

Left dorsal rib 10 (L10) has a smooth, inflated abnormality 35 mm below the tuberculum (Fig. 3). There is no visible offset, nor is there any indication of a fracture. The abnormality is demarcated from the non-pathological bone by an eye-shaped texture change 22 mm long (proximodistally) and spanning the width of the rib. The inflation expands the circumference of the bone from 55 mm distal to the abnormality to 67 mm at the widest point of the abnormality. On the anterior surface, there are two small erosive lesions with sharp edges that pass through the periosteal surface of the abnormality. The distal lesion is heart-shaped, 9 mm by 7 mm in size and 2**–**3 mm deep. The proximal lesion is smaller in size (5 mm by 5 mm) and is less than a millimeter deep (Fig. 3B). The lesions are located within the eye-shaped margin of the abnormality.

#### Diagnosis

Ribs L7 and R10 are classified as traumatic. The two rib fractures are consistent with direct trauma to the rib cage. Multiple aspects of these fractures suggest L7 and R10 are from the same traumatic event. L7 and R10 are both impacted fractures with 26 mm and 24 mm of compression, respectively, indicating an equivalent force fractured both ribs. Impacted fractures like this could only have occurred from a strong compressive force applied coincident to the long axis of the rib, like what would be expected from a direct fall to the ventral side. It is unlikely that an animal would sustain two injuries that produced such similar fracture morphologies, indicating that these pathologies are likely contemporaneous. Additionally, both ribs show similar degrees of callus formation. The ribs are clearly fused together, indicating some healing has occurred, and that these injuries occurred sometime prior to death.

Rib L10 is classified as traumatic-infectious. The presence of a callus or proliferative lesion is necessary but not sufficient to diagnose a pathology as traumatic^9^. Additional factors such as location and type of lesion need to be considered. The abnormality on L10 (Fig. 3A) is at the greatest curvature of the rib and the inflation is most prominent on the anterior side. The CT scans reveal no internal cortical bone and the surface of the inflation is smooth. These characteristics are consistent with a rib fracture in the later stages of callus remodeling^9,23^. The lytic lesions present on the anterior side of the callus are localized areas of bone destruction characteristic of osteomyelitis in reptiles and mammals^24,25^. The lesions present on the external surface of this callus suggest that there was either an infection in the surrounding soft tissue or that the infection had spread to the bone and was progressing into osteomyelitis.

### Metacarpal IV

**Figure 4:**
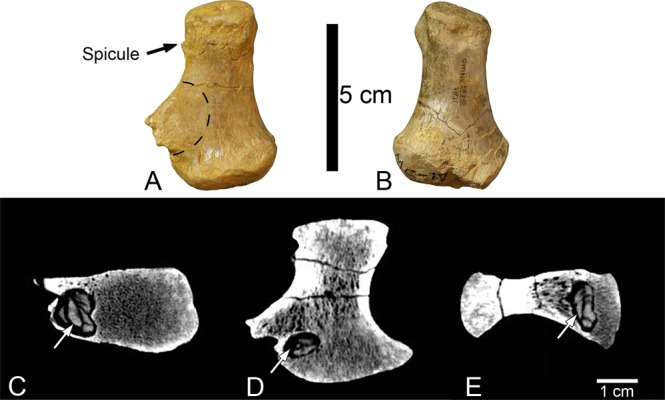
OMNH 58340 left and right metacarpal IV. (**A**) Pathological left metacarpal IV dorsal view, dashed line shows the location of a disruption in the periosteal surface texture, and the black arrow points to a 3 mm long projection on the proximal margin of the collateral ligament pit. (**B**) Right metacarpal IV dorsal view. (**C**–**E**) Computed tomography scans of left metacarpal IV showing the internal morphology consistent with a type of subacute pyogenic osteomyelitis called a Brodie abscess. (**C**) Proximal view (**D**). Dorsal view (**E**). Lateral view. Arrow indicates the presence of an irregular sequestrum within the abscess consistent with the morphology of a Brodie abscess in an early stage of its development.

A large osseous outgrowth is present 10 mm beyond the proximal articular surface on the lateral side of the diaphysis. The osseous outgrowth begins to diverge from the lateral side of the shaft 30 mm from the distal condyle forming a sub-triangular outgrowth that projects laterally 20 mm from the lateral side of the bone (Fig. 4A). The termination of the outgrowth is rugose and composed of cortical bone. There is no abnormal bone thickening in the dorsal and palmar directions; rather the outgrowth appears to continue the normal periosteal surface of the bone on the dorsal and palmar sides. However, on the dorsal side, there is a concentric disruption of the normal long grain bone texture, 20 mm in diameter (Fig. 4A), coinciding with an underlying intraosseous abscess (Fig. 4C–E). The texture within the disruption is completely disorganized. In addition to the large outgrowth, there is a small bone spicule 3 mm in length extending laterally from the proximal margin of the lateral collateral ligament pit. The proximal and distal epiphyses are unaffected.

Computed tomography (CT) scans of metacarpal IV were crucial in characterizing the internal morphology of this pathology. These CT scans reveal that adjacent to the osseous outgrowth there is an internal abscess that measures 11.5 × 9.2 × 6.2 mm (Length × Width × Height) that is ovoid in shape with the long axis oriented palmodorsally. The lesion is in the medullary cavity of the diaphysis 6 mm distal to the proximal condyle and within the cortical bone on the lateral side. The margin of the abscess is clear and distinct, with a dense rim of cancellous sclerosis circumscribing the abscess. Contained within the abscess is an irregularly-shaped material interpreted to be a sequestrum. It is unlikely that this material filling the abscess is a diagenetic mineral due to three reasons (1) the sequestrum mimics the abscess in shape, (2) the material has a density consistent with the surrounding bone, and (3) does not appear to originate from the perimeter of the abscess as would be expected from a precipitating mineral. Additionally, the osseous outgrowth is as it appears macroscopically, a well-organized outgrowth composed internally of cancellous bone and externally of cortical bone.

#### Diagnosis

The osseous outgrowth on metacarpal IV is interpreted as a response to the proliferation of an internal abscess and therefore is considered one pathology. The morphology of the metacarpal IV abnormality is consistent with the characteristics of a Brodie abscess, a type of subacute pyogenic osteomyelitis, therefore this pathology is classified as infectious. Brodie abscesses are variable in humans in terms of size, degree of osseous proliferative response, and location. Because of their variability, they are commonly misdiagnosed^26^. Their typical imaging appearance is characterized as an intraosseous bone lesion with an eccentric area of bone destruction that is variably emarginated by reactive sclerosis of the surrounding bone^26^. In a study of 25 confirmed cases of Brodie abscesses in humans, all but one occurred in the lower limbs, most commonly in the tibia and the femur^26^. Nine were present in the diaphysis and of those nine, four were in the medullary cavity. In all but four cases, cancellous sclerosis was present surrounding the abscess, and in ten of the cases, reactive growth like that of the outgrowth on metacarpal IV was present. Sequestrum was present in five of the 25 cases and the authors suggested that the presence of sequestra may indicate an abscess early in its development^26^.

A differential diagnosis including a Brodie abscess must also include a discussion of the characteristics of an osteoid osteoma—a type of benign bone tumor—and an osteochondroma as discussed above in reference to left phalanx I-1^27^. An osteoid osteoma produces a morphology that is similar to a Brodie abscess in CT scans, including a low attenuation nidus (hole) and adjacent sclerosis of the surrounding bone^27^. However, irregular sequestra like that seen within the abscess on the metacarpal IV of *Tenontosaurus* is not typical of an osteoid osteoma. Rather, a central osseous mineralization may be present within the nidus of the osteoid osteoma, but is spheroidal in shape^27,28^. The outgrowth present on this element exhibits the pathognomonic characteristic of an osteochondroma—continuity of the lesion with the underlying cortical and cancellous bone. However, again the presence of the internal abscess precludes this as a potential diagnosis, as abscesses are not commonly associated with osteochondromas. The abscess within metacarpal IV of OMNH 58340 exhibits many of the characteristics associated with Brodie abscesses, including; (1) a cancellous sclerotic rim that surrounds the area of bone destruction (Fig. 4C,D), (2) a reactive outgrowth closely associated with the abscess; and (3) an eccentric, irregularly shaped sequestrum typical of early-stage hematogenous osteomyelitis (Fig. 4C–E). Moreover, a diagnosis of this pathology is not unprecedented in non-avian dinosaurs. A Brodie abscess was described by Vittore and Henderson^28^ in the left pedal phalanx II-1 of the tyrannosaurid BMR P2002.4.1 (Burpee Museum of Natural History, Rockford, Illinois, USA). However, this is the first reported Brodie abscess in herbivorous dinosaurs.

## Discussion

The five pathologies diagnosed herein for the skeleton of OMNH 58340 are classified sensu Hanna^11^: left dorsal rib 7 (L7) and right dorsal rib 10 (R10) are traumatic, the left pedal phalanx I-1 is traumatic—and possibly infectious—left dorsal rib 10 (L10) is traumatic-infectious, and left metacarpal IV is infectious. The pathologies left phalanx I-1, L7, L10, and R10 appear to be in the later stages of callus healing, suggesting they may be contemporaneous injuries. While it is difficult to know the cause of these injuries, the pathologies on the ribs and phalanx I-1 are consistent with injuries that may have resulted from a fall, possibly a consequence of inter- or intraspecific interactions. The development of the Brodie abscess is difficult to constrain directly; the symptoms associated with a Brodie abscess have been reported to last from four weeks to ten years in humans and have not been shown to correlate with trauma^26^. Therefore it is possible, the Brodie abscess preceded all other injuries present on OMNH 58340, or the abscess could have developed following the fall, ultimately, the timing of this injury is equivocal^26,29^.

All pathologies present show signs of healing indicating that these injuries were not immediately fatal, yet the complications associated with these injuries may have contributed to this individual’s death. The fractures present on phalanx I-1 and L10 would have caused localized swelling and chronic pain, although the post-traumatic osteomyelitis likely had greater systemic effects. Fighting infection such as osteomyelitis requires a strong immune response, thus demanding an increased intake of food and fluids^12^. The symptoms of a Brodie abscess are generally isolated to the affected limb and include a dull recurrent ache, local swelling, and in some patients a pronounced limp^26,29^. Therefore, the Brodie abscess present in metacarpal IV, in combination with the pathological phalanx, may have inhibited this animal’s ability to locomote and acquire food resulting in malnutrition and a suppressed immune system, potentially leaving it susceptible to greater secondary infection^12^.

Osteomyelitis such as a Brodie abscess forms when pyogenic bacteria enters the bone via three possible vectors: (1) transmission through the infection of adjacent soft tissue, (2) direct transmission into the bone (e.g. through a compound fracture) and (3) transmission through the bloodstream (hematogenously) from another septic source^25^. While the condition of the soft tissue adjacent to the metacarpal IV is unknown, metacarpal IV shows no evidence of trauma, therefore direct transmission due to injury is unlikely. In humans, hematogenous transmission is the most common vector of development and therefore is considered the most likely method of transmission for this pathology^26^. In many cases, the septic source for a Brodie abscess cannot be identified. In a case study of 25 Brodie abscesses only 5 patients reported prior instances of infection varying from a staphylococcal abscess of the arm to blood poisoning^26^. Other suggested septic sources include skin abscesses, infections of the genitourinary, gastrointestinal, biliary, and respiratory systems^28^. Therefore, antecedent infection in the soft tissues should always remain a possible septic source of bacteria when looking at sources of infection in the fossil record. In the only other reported case of a Brodie abscess in non-avian dinosaurs, in which concomitant infected elements were lacking on the remains of a tyrannosaurid BMR P2002.4.1, Vittore and Henderson^28^ suggested that dental trauma may have been the septic source of bacteria for the Brodie abscess on left phalanx II-1. The most likely septic source for the Brodie abscess in OMNH 58340 is the infections present in phalanx I-1 and rib L10. However, because the state of soft tissue in this individual cannot be assessed, infection in the soft tissues cannot be ruled out. Therefore, the Brodie abscess may have preceded the trauma related injuries.

Multiple studies have shown the utility of micro-CT and CT scanning as a non-invasive way of studying the internal morphology of bones^22,28,30,31^. The application of CT scanning in this diagnosis was integral for narrowing the range of possible etiologies. The identification of the original cortical bone within the callus on phalanx I-1 was crucial in differentiating between a diagnosis of an osteochondroma—which had been suggested for similar structures on MOR 693 and UUVP 1657—and a callus with a post-traumatic infection^11^. Furthermore, prior to CT scanning, the morphology of the metacarpal IV abnormality was consistent with tendon avulsion, stress fracture, osteochondroma, and enthesopathy. The visualization of an internal abscess through CT reduced the differential diagnosis to three possibilities: a Brodie abscess, an osteoid osteoma, and an osteochondroma. The CT scans revealed that the pathology was most consistent with a Brodie abscess, confirming the second report of hematogenous osteomyelitis in non-avian dinosaurs and the first report in Ornithopoda. Future study methods should include CT scanning as a diagnostic tool, especially if the pathological elements in question are located in the axial skeleton, where hematogenous osteomyelitis is most likely to occur^25,26,28,32^.

The presence of hematogenous osteomyelitis, in the form of a Brodie abscess, in this individual and BMR P2002.4.1 suggests that the hematogenous spread of infection in dinosaurs may be more common than previously recognized^28^. Foth, *et al*.^4^ used the lack of hematogenous osteomyelitis and the isolation of infection to single elements in *Allosaurus* to suggest that extant phylogenetic bracketing should be applied in pathological studies and a reptilian-immune response should be considered for dinosaurs, while mammalian comparisons should be avoided. This is based on the differing physiological responses to infection present in mammals, birds, and reptiles. Upon infection by pyogenic organisms, reptiles and birds produce a caseous substance called fibrin. If the infection continues or does not rupture to the surface, a hard, encapsulating mass forms around the focus of infection, called a fibriscess^33,34^. The isolation of a pathogen in a fibriscess appears to restrict the spread of infection, reducing the likelihood of septicemia in birds and reptiles^33^. In contrast, mammals produce pus at the focus of an infection, forming a suppurative abscess by which adjacent tissues are more likely to be affected, with an increased chance of septicemia relative to reptiles^25^. The pathologies on OMNH 58340 do not show dissemination to adjacent bones, consistent with the observation of infected bones in *Allosaurus* and other dinosaurs, yet the Brodie abscess is most likely hematogenous in origin^4,6,9,11,12^. The presence of hematogenous osteomyelitis may indicate that a more avian-like immune response to infection should be considered for dinosaurs because, although rare in birds, birds do exhibit hematogenous osteomyelitis^32,35^.

When describing pathologies, previous investigators have chosen to use a mammalian model, particularly in reference to infection^7,9,11,12,28^. This is likely due to two reasons: (1) there is abundant literature on mammalian pathologies owing to an anthropocentric bias in medical literature, and (2) infectious pathologies in dinosaurs bear a strong resemblance to mammalian infectious pathologies. Rega^9^ noted that the morphology of an abnormality on the fibula of a *Tyrannosaurus rex*, FMNH PR 2018 (Field Museum of Natural History, Chicago, Illinois, USA) has an involucrum, sequestrum, and cloaca, all suggestive of a mammalian pus producing reaction. However, the described morphology does not necessitate that dinosaurs produced pus. Rather, it suggests that the response to osteomyelitis in dinosaurs and mammals might produce the same osseous morphology. Two studies—Emslie and Nade^31^, and Tully, *et al*.^36^—noted that osteomyelitis in birds follows a similar pathogenesis to mammals and can produce similar osseous responses in analogous elements. Tully, *et al*.^36^ described osteomyelitis in the tarsometatarsus of two ratites, both of which exhibit a sequestrum and involucrum. The radiographs of an ostrich show an involucrum, sequestrum, and a small sinus tract (cloaca) draining liquid exudate through a fistula in the skin, characters commonly associated with mammalian osteomyelitis. Tully, *et al*.^36^ noted that the presence of sequestra in ratites bears a strong resemblance to sequestra commonly found in infected metacarpals and metatarsals of horses. These examples indicate that mammalian descriptors and diagnoses might not be wholly inapplicable when describing dinosaurian infection as some researchers have suggested^4,6^. The similarity in osseous response to infection in birds and mammals should be the subject of future studies to better inform descriptions of infectious pathologies in dinosaurs. Owing to the similarities between birds and mammals discussed above, and the similarities in morphology of our specimen with the characteristics of a mammalian Brodie abscess, we chose to use mammalian descriptors when diagnosing the internal abscess present in metacarpal IV. We implore future researchers to consider use of mammalian descriptors when describing infection if such descriptors appear to accurately describe the morphology of the infection.
